# ShRNA-Targeted Centromere Protein A Inhibits Hepatocellular Carcinoma Growth

**DOI:** 10.1371/journal.pone.0017794

**Published:** 2011-03-15

**Authors:** Yongmei Li, Zhi Zhu, Shuhui Zhang, Danghui Yu, Hongyu Yu, Lina Liu, Xiaozhe Cao, Li Wang, Hengjun Gao, Minghua Zhu

**Affiliations:** 1 Department of Pathology, Changhai Hospital, Second Military Medical University, Shanghai, China; 2 Institute of Liver Diseases, Second Military Medical University, Shanghai, China; 3 Department of Pathology, Yueyang Hospital, Shanghai University of Traditional Chinese Medicine, Shanghai, China; 4 Department of Pathology, Medical College, Tongji University, Shanghai, China; 5 National Engineering Center for Biochip at Shanghai, Shanghai, China; Deutsches Krebsforschungszentrum, Germany

## Abstract

**Background:**

Centromere protein A (CENP-A) plays important roles in cell-cycle regulation and genetic stability. Herein, we aimed to investigate its expression pattern, clinical significance, and biological function in hepatocellular carcinoma (HCC).

**Methodology/Principal Findings:**

CENP-A expression at the mRNA and protein levels was examined in 20 pairs of fresh HCCs and corresponding nontumor liver tissues. Immunohistochemistry for CENP-A was performed on 80 paraffin-embedded HCC specimens, and the clinical significance of its expression was analyzed. A human HCC cell line HepG2 with high abundance of CENP-A was used to study the effects of manipulating CENP-A on HCC growth. Quantitative real-time polymerase chain reaction arrays and Western blot analysis were employed to identify the cell-cycle control- and apoptosis-related genes regulated by CENP-A. The results showed that CENP-A was aberrantly overexpressed in HCCs relative to adjacent nontumor tissues. This overexpression was significantly associated with positive serum HBsAg status, increased histological grade, high Ki-67 index and P53 immunopositivity. Knockdown of CENP-A in HepG2 cells reduced cell proliferation, blocked cell cycle at the G1 phase, and increased apoptosis. The antiproliferative effects of CENP-A silencing were also observed *in vivo*. Conversely, CENP-A overexpression promoted HCC cell growth and reduced apoptosis. Furthermore, many genes implicated in cell-cycle regulation and apoptosis, including CHK2, P21waf1, P27 Kip1, SKP2, cyclin G1, MDM2, Bcl-2, and Bax, were deregulated by manipulating CENP-A.

**Conclusions/Significance:**

Overexpression of CENP-A is frequently observed in HCC. Targeting CENP-A can inhibit HCC growth, likely through the regulation of a large number genes involved in cell-cycle progression and apoptosis, and thereby represents a potential therapeutic strategy for this malignancy.

## Introduction

Hepatocellular carcinoma (HCC) is one of the most prevalent malignancies in the world, with a particularly high incidence in Asian countries [1], [2]. Epidemiological studies reveal that chronic hepatitis B and C viral infection, chronic alcohol consumption, and ingestion of aflatoxin B1-contaminated food are important aetiological factors of HCC [1], [3]. Although a wide range of genetic and epigenetic alterations have been identified contributing to the deregulation of key oncogenes and tumor-suppressor genes, genetic events in hepatic carcinogenesis are poorly understood [1], [2].

Genome stability relies on the accurate partitioning of chromosomes into the daughter cells during division. Centromeres are the chromatin regions associated with kinetochores, which mediate chromosome segregation and the mitotic checkpoint [4]–[6]. The kinetochore is assembled specifically during mitosis on a specialized region of each chromosome called the centromere, which is constitutively bound by >15 centromere-specific proteins [7]–[9]. Centromere protein (CENP)-A, a centromere-specific 17-kDa protein, is one of the first identified kinetochore components in humans. It is a unique histone H3-like protein only found in active centromeres and believed to be a central element in the epigenetic maintenance of centromere identity [9]–[15]. CENP-A is well conserved among eukaryotes; loss of its function due to mutations or deletions results in chromosome missegregation, suggesting its requirement for the maintenance of genetic stability [16]–[18]. Accumulating evidence indicates that CENP-A and other centromere proteins are frequently upregulated in cancers and their overexpression is associated with the development of aneuploidy, a hallmark of malignant cells [16], [19]–[23]. Blocking CENP-H expression using RNA interference (RNAi) technology significantly decreased mitotic index and prolonged cellular doubling time [24]. CENP-E silencing in HeLa cells leads to missegregation of chromosomes after a mitotic delay and produces aneuploid daughter cells [25]. These observations suggest that the aberrant expression of the CENP family members may be closely linked to carcinogenesis.

Our previous works have shown that CENP-A was overexpressed in HCC [26], and RNAi-mediated depletion of CENP-A in one HCC cell line HepG2 caused a cell cycle arrest at the G1 phase [27]. In the present study, we aimed to confirm the effects of manipulating CENP-A on HCC growth and to clarify the underlying molecular mechanism for CENP-A action. Additionally, the clinical significance of CENP-A expression in HCC was evaluated.

## Results

### Overexpression of CENP-A in human HCC cell lines and tumor tissues

**Figure 1:**
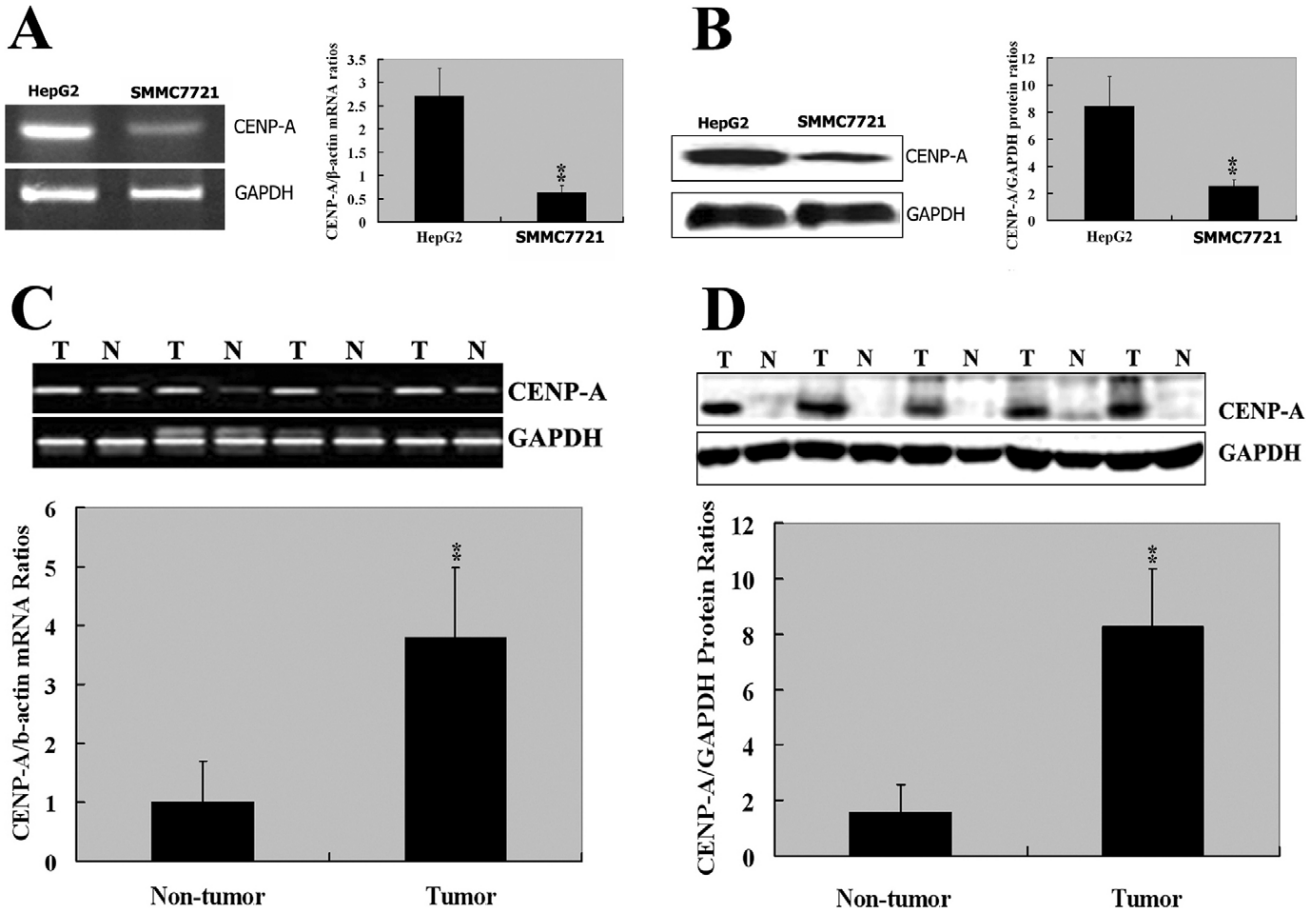
CENP-A mRNA and protein expression in HCC cell lines and tumor tissues. **A**: RT-PCR (left) and qPCR (right) analysis of CENP-A mRNA expression in two HCC cell lines HepG2 and SMMC-7721. **B**: Western blotting analysis of CENP-A protein product in HepG2 and SMMC-7721 cells. Left: Representative Western blots of three independent experiments. Right: Quantification of the CENP-A Western blots. **C**: RT-PCR (upper) and qPCR (lower) analysis of CENP-A transcript in HCC tumor (T) and corresponding nontumorous (N) liver tissues. **D**: Western blotting analysis of CENP-A protein expression in HCC and adjacent nontumor tissues. The bar graph below the Western blot panels shows the quantification of the protein level of CENP-A. GAPDH was used as an internal control in all the experiments. ^**^ *P*<0.01 using Student's *t*-test; *n* = 3 in A and B, and *n* = 20 in C and D.

Analysis of CENP-A transcript using a real-time quantitative polymerase chain reaction (qPCR) assay showed that HepG2 cells expressed a significantly higher level of CENP-A mRNA than did another HCC cell line SMMC-7721 (*P*<0.01; Figure 1A). Western blotting analysis further revealed that the CENP-A protein level was significantly higher in HepG2 cells compared to that in SMMC-7721 (*P*<0.01; Figure 1B). Given the high abundance of endogenous CENP-A, HepG2 cells were used in the following knockdown experiments. We also examined the expression of CENP-A in 20 pairs of snap-frozen HCC and adjacent nonmalignant liver tissues. The CENP-A mRNA and protein levels were significantly (*P*<0.01) elevated in HCC specimens compared to the corresponding nontumor tissues, as shown in Figure 1C&1D, respectively.

### Associations between CENP-A expression and clinicopathologic parameters of HCC patients

**Figure 2:**
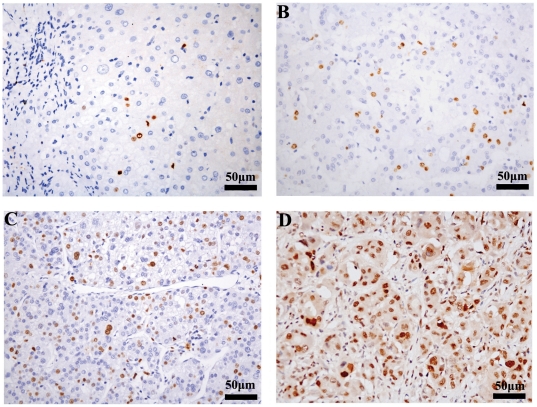
Immunohistochemistry for CENP-A in HCC and adjacent nontumor liver tissues. Representative CENP-A staining in (**A**) nontumorous liver tissue, and in (**B**) well-differentiated, (**C**) moderately-differentiated, and (**D**) poorly-differentiated HCC. The CENP-A staining is predominantly nuclear in the samples examined, with concurrent diffuse cytoplasmic staining in poorly-differentiated HCC.

Immunohistochemistry using anti-CENP-A antibody was performed on a set of 80 pairs of paraffin-embedded human HCC specimens and adjacent nontumorous liver tissues. A low frequency of the surrounding nontumorous tissues (35/80, 44%) displayed rare positive nuclei (Figure 2A), while a markedly higher proportion of HCC samples (57/80, 71%) showed positive CENP-A immunostaining (Figure 2B–D). Expression of CENP-A appeared to increase with the grades of differentiation of HCC cells from well differentiated to poorly differentiated (Figure 2B–D). Moreover, the CENP-A immunostaining pattern varied among the tumors with different degree of differentiation. Well- and moderately-differentiated HCC cells had an exclusive nuclear staining pattern for CENP-A (Figure 2B&2C), while poorly-differentiated cells displayed a mixed staining pattern, with intense nuclear staining and weak, diffuse cytoplasmic staining (Figure 2D).

Next, we investigated the relationship between CENP-A expression and clinicopathologic parameters of HCC patients. As shown in Table 1, a higher frequency of CENP-A overexpression was found in serum HBsAg positive patients (44/65, 68%) than in negative ones (2/15, 13%; *P* = 0.014). There was an increasing tendency for CENP-A expression with the progression of histological grade (*P* = 0.017, I versus II; *P* = 0.021, I versus III; *P* = 0.004, I versus IV). Additionally, CENP-A expression was positively correlated with the Ki-67 staining index that was defined as the percentage of Ki-67 immunopositive cells (*r* = 0.685, *P*<0.001). There was a significant relationship between CENP-A and P53 at the protein level (*r* = 0.72, *P*<0.001), but no correlation was noticed between CENP-A and P21waf1 protein levels.

**Table 1:** Clinicopathologic significance of CENP-A overexpression in HCC (n = 80).

Variables	No.	CENP-A immunoreactivity			*P*
		Negative	Low	High	
Sex					
Male	61	19	8	34	0.190
Female	19	4	3	12	
Age					
Less than median	38	10	7	21	0.645
Greater than median	42	13	4	25	
Serum AFP level (µg/l)					
<20	38	9	6	23	0.517
≥20	42	14	5	23	
Serum HBsAg					
Positive	65	17	4	44	0.014
Negative	15	6	7	2	
Tumor size					
≤2 cm	28	8	5	15	0.553
>2 cm	52	15	6	31	
Histological grades					
I	10	7	2	1	
II	34	9	8	17	0.017
III	28	7	0	21	0.021
IV	8	0	1	7	0.004
Liver cirrhosis					
Absent	21	5	4	12	0.621
Present	59	18	7	34	
Tumor capsule					
Intact	20	7	4	9	0.193
Absent or not intact	60	16	7	37	
Intrahepatic metastasis					
Not observed	28	10	4	14	0.683
Observed	52	13	7	32	
P53					
Positive	60	6	54	<0.001	
Negative	20	17	3		
Ki-67 index					
≥50%	43	0	43	<0.001	
<50%	37	23	14		
P21^waf1^					
Positive	25	5	20		
Negative	55	18	37	0.224	
Total	80	23	11	46	

### Alteration of CENP-A expression affects cell growth and apoptosis of HepG2 cells

**Figure 3:**
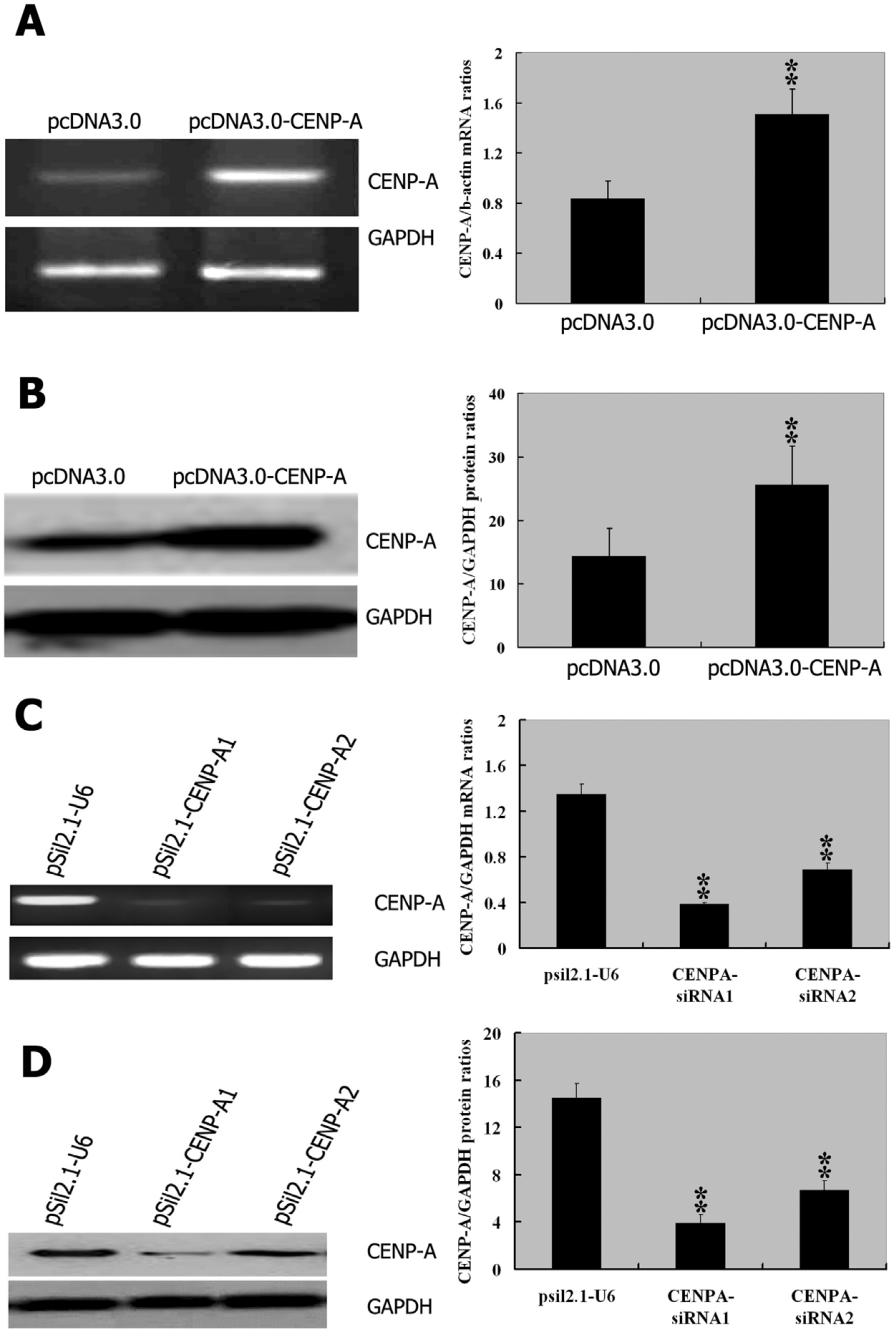
CENP-A overexpression and knockdown in HepG2 cells. Analysis of CENP-A mRNA (**A**, **C**) and protein (**B**, **D**) levels in HepG2 cells transfected with CENP-A-expressing plasmids or siRNA-expressing plasmids, as described in Materials and methods. **A**: RT-PCR (left) and qPCR (right) measurement of CENP-A transcript in cells transfected with pcDNA3.0-CENP-A or pcDNA3.0 empty vector. ^**^ *P*<0.01 using Student's *t*-test; *n* = 3. **B, left**: Representative Western blots for CENP-A in cells treated as in A. Right: Quantification of the blotting shown in the left panel. ^**^ *P*<0.01 using Student's *t*-test; *n* = 3. **C**: RT-PCR (left) and qPCR (right) examination of CENP-A mRNA levels in HepG2 cells transfected with CENP-A1, CENP-A2, or pSil2.1-U6 empty vector (control). ^**^ *P*<0.01 compared to cells with the pSil2.1-U6, using one-way ANOVA; *n* = 3. **D**: Immunoblot of cells treated as in C. Left: Representative Western blots for CENP-A. Right: Quantification of the blotting shown in the left panel. ^**^ *P*<0.01 compared to cells with the pSil2.1-U6, using one-way ANOVA; *n* = 3. GAPDH was used as an internal control in all the experiments.

To elucidate the biological function of CENP-A in HCC, we overexpressed and knocked down this gene in HepG2 cells. The delivery of CENP-A-expressing constructs (pcDNA3.0-CENP-A) resulted in a significant elevation in CENP-A expression at both mRNA and protein levels, as compared to the control cells transfected with pcDNA3.0 empty vector (*P*<0.01; Figure 3A&3B). Conversely, the stable transfection of CENP-A small interfering RNAs (siRNAs) significantly diminished the endogenous CENP-A expression levels of HepG2 cells (*P*<0.01 versus mock transfectants; Figure 3C&3D). The CENP-A1 siRNA appeared to be more efficient in targeting CENP-A than CENP-A2, resulting in a reduction by more than 70% in the CENP-A mRNA and protein amounts.

**Figure 4:**
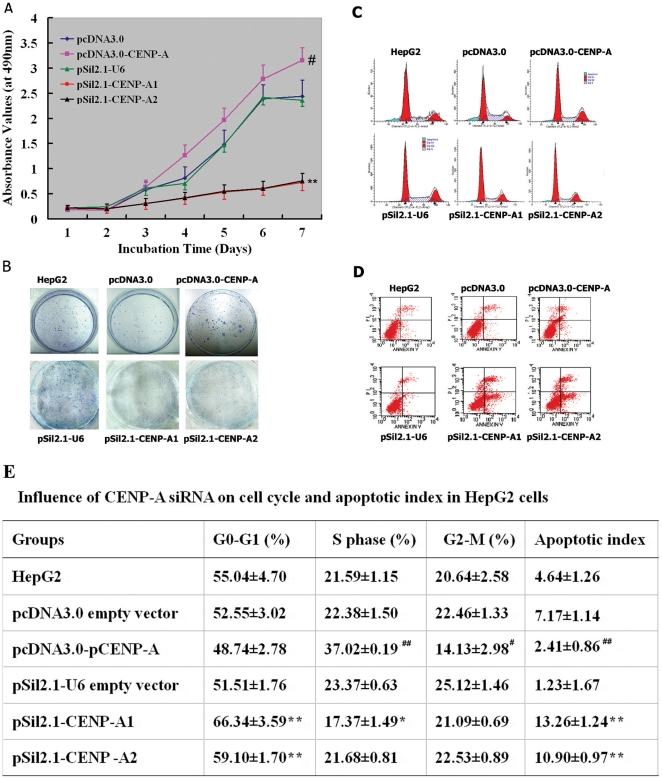
Effects of manipulating CENP-A on cell proliferation and apoptosis in HepG2 cells. HepG2 cells were transfected with CENP-A-expressing plasmids, siRNA-expressing plasmids, or empty vectors, as described in Materials and methods. The stable transfectants were subjected to the following experiments. **A**: Cell proliferation assay. The HepG2 transfectants were seeded in 96-well microplates in sextuplicate and cultured for up to 7 days. Cell proliferation was assessed by the MTT assay. ^**^ *P*<0.01 between pSil2.1-U6 and pSil2.1-CENP-A1 or pSil2.1-CENP-A2; ^#^ *P*<0.05 between pcDNA3.0 and pcDNA3.0-CENP-A; *n* = 3. **B**: Colony formation assay. The transfected or untreated HepG2 cells were plated in 6-well plates at a density of 1,000 cells per well. After 10 days, cells were stained with Giemsa and colonies consisting of >50 cells were scored. Representative dishes of three independent experiments are shown. **C–E**: Cell-cycle and apoptosis analysis. The transfected or untreated HepG2 cells were stained with PI alone or FITC-annexin V and PI and analyzed by flow cytometry. Representative histograms (C) and dot plots (D) of three independent experiments are shown. Apoptotic cells in D are FITC-annexin V positive and PI negative. **E**: Quantification of the cell-cycle and apoptosis analysis. The apoptotic index was determined as the ratio of apoptotic cell number to total cell number. ^#^ *P*<0.05, ^##^ *P*<0.01, compared to the pcDNA3.0 empty vector; ^*^ *P*<0.05, ^**^ *P*<0.01, compared to the pSil2.1-U6; *n* = 3.

When compared to the pcDNA3.0 control cells, CENP-A overexpression significantly increased cell proliferation assessed by 3-(4,5-dimethylthiazol-2-yl)-2,5-diphenyltetrazolium bromide (MTT) assay (P<0.05; Figure 4A). Colony formation assay revealed a markedly increased colony number and size by the overexpression of CENP-A (Figure 4B). A promotion of the cell cycle from the G0/G1 to S phase was observed in the pcDNA3.0-CENP-A cells (Figure 4C &4E). Moreover, apoptosis analysis using annexin V and propidium iodide (PI) staining showed that the pcDNA3.0-CENP-A cells had a significantly lower apoptotic index than the pcDNA3.0 control cells (2.41±0.86% versus 7.17±1.14%, *P*<0.01; Figure 4D &4E). Converse to CENP-A overexpression, its depletion by siRNAs significantly (*P*<0.05) inhibited cell proliferation and colony formation compared to the pSil2.1-U6 mock transfectants (Figure 4A&4B). Moreover, CENP-A silencing caused a cell cycle arrest at the G1 phase and a significant increase in apoptosis (P<0.01 compared to the pSil2.1-U6 transfectants; Figure 4C–E).

### Effects of manipulating CENP-A on tumor growth in a HepG2 xenograft model

**Figure 5:**
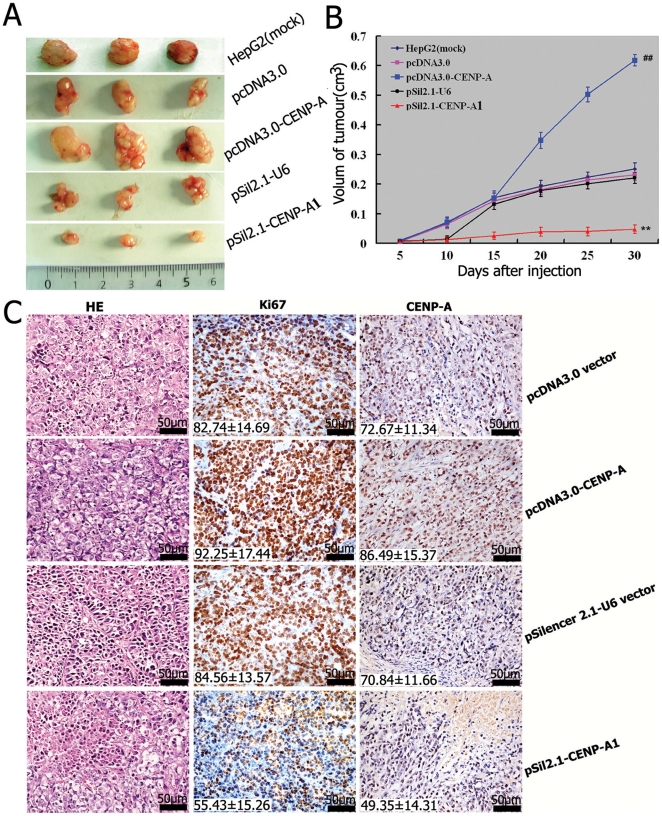
Effects of CENP-A overexpression or knockdown on tumorigenicity in nude mice. The transfected or untreated HepG2 cells were injected into nude mice, as described in Materials and methods. Tumor volume was determined every 5 days for 30 days. At the end of the experiment, animals were sacrificed and tumors were excised for volume measurement and histological study. **A**: Representative photographs of tumors for each treatment. **B**: Growth curve of tumor xenografts. ^##^ *P*<0.01, compared to the pcDNA3.0 empty vector; ^**^ *P*<0.01, compared to the pSil2.1-U6; *n* = 4. **C**: The resected tumors were stained with HE and antibodies against Ki-67 and CENP-A. Representative images of the staining are shown. The percentages of Ki-67-positive and CENP-A-positive cells were inserted in the corresponding images.

**Figure 6:**
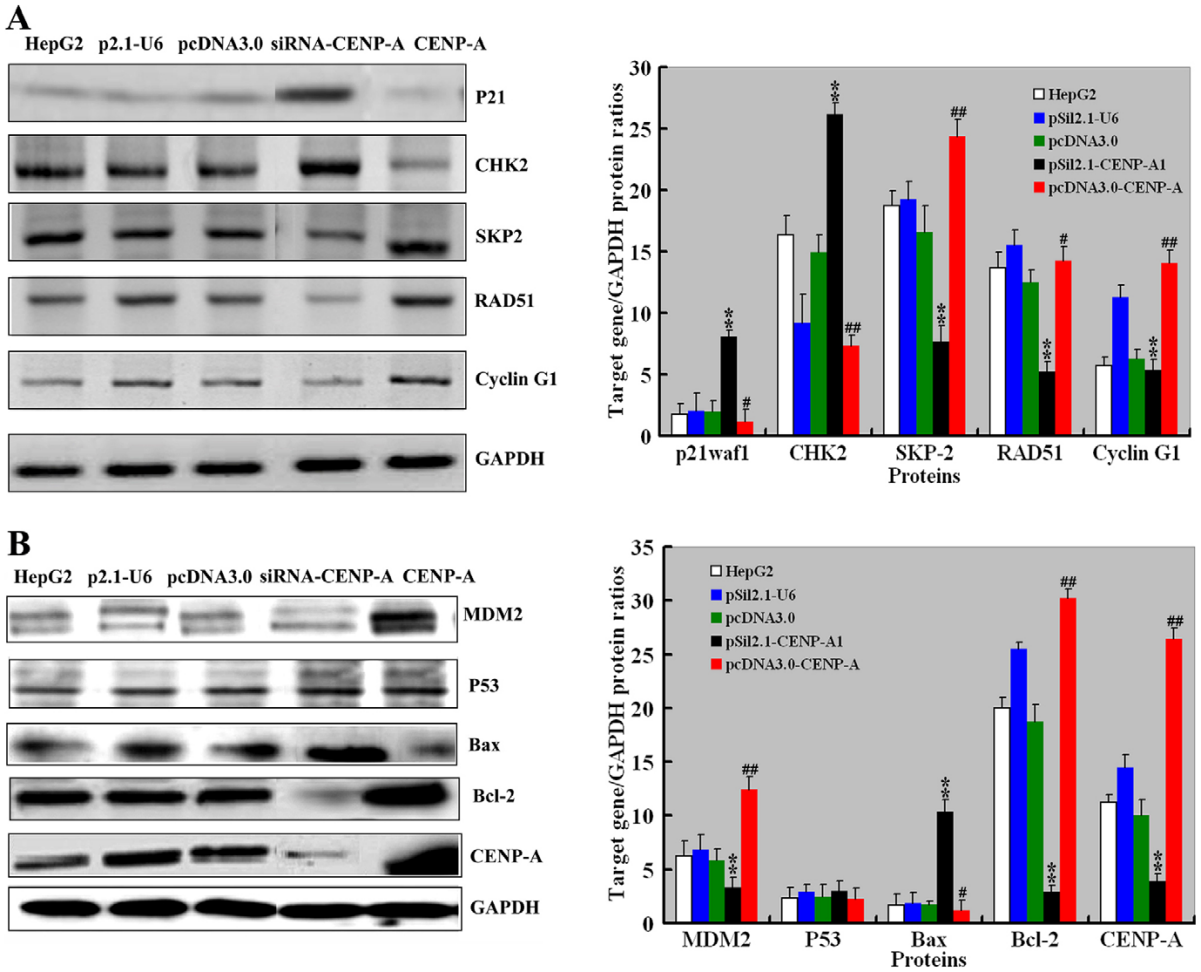
Alteration of gene expression profiling by CENP-A overexpression or knockdown in HepG2 cells. HepG2 cells were untreated or transfected with CENP-A-expressing plasmids, siRNA-expressing plasmids, or empty vectors (pcDNA3.0, and pSil2.1-U6). Forty-eight hours after the transfection, cells were subjected to Western blotting for the proteins indicated. **A&B**: Representative Western blots are shown in left panels. Right panels show the quantification of the immunoblots. ^#^ *P*<0.05, ^##^ *P*<0.01, compared to the pcDNA3.0 empty vector; ^**^ *P*<0.01, compared to the pSil2.1-U6; *n* = 3.

Next, we checked whether manipulating CENP-A affected tumor growth in vivo, using a HepG2 xenograft model. Measurement of tumor volumes showed that the cells overexpressing CENP-A formed significantly larger tumors than the control cells with pcDNA3.0 empty vector (*P*<0.01; Figure 5A&5B). Conversely, downregulation of CENP-A by CENP-A1 resulted in a significant suppression of tumor growth as compared with the delivery of the pSil2.1-U6 mock vector (*P*<0.01). Immunohistochemistry for CENP-A confirmed increased staining in the pcDNA3.0-CENP-A tumors compared with pcDNA3.0 vector tumors (Figure 6C). The pSil2.1-CENP-A1 tumors, as expected, had reduced immunoreactivity for CENP-A relative to the pSil2.1-U6 tumors. Most interestingly, the Ki-67 immunoreactivity varied in parallel with the alteration of CENP-A staining; i.e., the Ki-67 staining index was elevated in the pcDNA3.0-CENP-A tumors in comparison with the pcDNA3.0 tumors (92.25±17.44% versus 82.74±14.69%, *P*<0.05) and lowered in the pSil2.1-CENP-A1 tumors relative to the pSil2.1-U6 tumors (55.43±15.26% versus 84.56±13.57%, *P*<0.01). These results collectively indicate an important role for CENP-A in the regulation of HCC growth.

### Modulation of the expression of cell growth and apoptosis related genes by CENP-A in HepG2 cells

To get more insight into the mechanism of CENP-A action, we employed a real-time PCR array to profile the expression of genes involved in the cell-cycle regulation. The gene expression profiling was analyzed in the pSil2.1-CENP-A1 and pSil2.1-U6 (control) transfectants. Of the 92 genes examined (Supplementary Table S1), 20 showed a >2-fold change in expression, 3 upregulated and 17 downregulated (Table 2). Interestingly, all the 3 upregulated genes (i.e., *CHK2*, *P21waf1*, and *P27 Kip1*) are negative regulators of cell cycle [28]–[30]. Among the most downregulated genes were SKP2, RAD51, and CCNG1, whose function is related to cell-cycle progression [31]–[33]. To confirm the findings using the real-time PCR array, Western blot analysis was performed to check the changes of protein levels. CHK2 and P21waf1 protein levels were significantly increased by CENP-A silencing, while SKP2, RAD51, and CCNG1 were decreased under the same condition (*P*<0.01 versus the pSil2.1-U6-transfected cells; Figure 6A). Additionally, CENP-A overexpression resulted in a significant decrease in the CHK2 and P21waf1 protein levels and elevation in the levels of SKP2, RAD51, and CCNG1 (*P*<0.01 versus the transfection of pcDNA3.0). Taken together, the deregulation of cell-cycle-related genes partially explains the CENP-A-depletion-induced growth arrest.

**Table 2:** Gene expression altered by CENP-A knockdown in HepG2 cells (>2-fold change).

Description	Genes title	Genebank ID	Abbreviation	Cytoband	Fold Difference Ratio
G1 phase and G1/S transition	S-phase kinase-associated protein 2 (p45)	NM_005983	SKP2	5p13	0.22±0.05
S phase and DNA replication	Minichromosome maintenance complex component 5	NM_006739	MCM5	22q13.1	0.35±0.07
	Minichromosome maintenance complex component 3	NM_002388	MCM3	6p12	0.37±0.05
	Minichromosome maintenance complex component 4	NM_005914	MCM4	8q11.2	0.42±0.09
G2 phase and G2/M transition	Cyclin G1	NM_004060	CCNG1	5q32-q34	0.27±0.06
	DIRAS family, GTP-binding RAS-like 3	NM_004675	DIRAS3	1p31	0.35±0.04
	DEAD/H (Asp-Glu-Ala-Asp/His) box polypeptide 11 (CHL1-like helicase homolog, S. cerevisiae)	NM_004399	DDX11	12p11	0.36±0.06
	Cyclin B1	NM_031966	CCNB1	5q12	0.38±0.08
	Baculoviral IAP repeat-containing 5 (survivin)	NM_001168	BIRC5	17q25	0.39±0.05
	Cyclin T2	NM_001241	CCNT2	2q21.3	0.46±0.09
	Cyclin-dependent kinase 2	NM_001798	CDK2	12q13	0.47±0.04
	Cyclin-dependent kinase 5, regulatory subunit 1 (p35)	NM_003885	CDK5R1		0.49±0.07
Cell cycle checkpoint and cell cycle arrest	Cyclin-dependent kinase inhibitor 1A (p21, waf1)	NM_000389	CDKN1A	6p21.2	8.33±0.13
	CHK2 checkpoint homolog (S. pombe)	NM_007194	CHEK2	22q12.1	4.55±0.08
	RAD1 homolog (S. pombe)	NM_002853	RAD1	5p13.2	0.43±0.07
	RAD51 homolog (RecA homolog, E. coli) (S. cerevisiae)	NM_002875	RAD51	15q15.1	0.21±0.09
	Cyclin-dependent kinase inhibitor 1B (p27, Kip1)	NM_004064	CDKN2B	9p21	2.08±0.05
Regulation of cell cycle	Transcription factor Dp-1	NM_007111	TFDP1	13q34	0.31±0.04
	Cyclin C	NM_005190	CCNC	6q21	0.45±0.07
Negative regulation of cell cycle	Retinoblastoma-like 1 (p107)	NM_002895	RBL1	20q11.2	0.46±0.08

The P53 pathway has been recognized to be important for cell-cycle arrest and apoptosis [34]. We next examined some key components of this pathway, including P53, MDM2 (an endogenous inhibitor of P53 [35]), anti-apoptotic protein Bcl-2, and pro-apoptotic protein Bax [36], in the HepG2 cells treated as above. The protein levels of MDM2 and Bcl-2 were significantly elevated by CENP-A overexpression (P<0.01; Figure 6B). An opposing trend was observed for Bax expression in CENP-A-overexpressing cells. In the setting of CENP-A silencing, MDM2 and Bcl-2 amounts were significantly lowered while Bax expression was potentiated. However, no significant difference was detected in the P53 protein level when the CENP-A level was altered.

## Discussion

In this study, we confirm and extend our previous findings [26] that CENP-A expression is significantly elevated in HCC. Moreover, the protein product of CENP-A is increased in parallel with its transcript, suggesting that CENP-A overexpression occurs at the transcriptional level. Clinical association analysis reveals that CENP-A overexpression is positively correlated with the Ki-67 index and the tumor histological grade, implying its involvement in HCC development. To dissect the function of CENP-A in HCC, we employed gain- and loss-of-function approaches to assess the effects of manipulating CENPA-A on cell growth and apoptosis in HepG2 cells. Overexpression of CENP-A significantly increased the cell proliferation and colony number in vitro, while conversely, depletion of its expression resulted in a marked decrease in cell growth. Using a HepG2 xenograft model in nude mice, we found that manipulating CENP-A also significantly affected tumor growth in vivo. These results highlight a crucial role for CENP-A in the regulation of HCC growth.

CENP-A plays a central role in directing kinetochore assembly, which is indispensable for equal chromosome segregation. Régnier and colleagues [17] have reported that CENP-A disruption impairs kinetochore formation in the chicken DT40 cell line and CENP-A-depleted cells proceed through anaphase and cytokinesis with unequal chromosome segregation. By depleting the Drosophila homolog of CENP-A, CID, Blower and colleagues [37] showed that CID was required for normal kinetochore formation and function, as well as cell-cycle progression. A recent study by Maehara et al. [38] has indicated that CENP-A reduction in primary human diploid lung fibroblasts induces a P53-dependent cellular senescence, an irreversible growth arrest. Consistent with the previous findings, our data demonstrate that RNAi-mediated knockdown of CENP-A causes a cell cycle arrest at the G1 phase and increases apoptosis in HepG2 cells. Converse to the inhibitory effects of CENP-A depletion, overexpression of CENP-A significantly promotes cell-cycle progression and favors cell survival. These results collectively suggest that aberrant expression of CENP-A may be an important etiologic factor for the pathogenesis of HCC. Indeed, CENP-A overexpression has been proposed to contribute to the aneuploidy of tumors [17], [39].

To clarify the molecular mechanism by which CENP-A affects cell proliferation, we employed the real-time PCR array to profile the expression of cell cycle-related genes. Three of the 20 genes altered >2 fold were upregulated when the CENP-A expression was knocked down. The 3 upregulated genes (CHK2, P21waf1, and P27 Kip1) play a negative role in cell-cycle progression [28]–[30]. The cyclin-dependent kinase (CDK) inhibitors P21waf1 and P27 Kip1 can bind to and inhibit the activity of various cyclin-CDK complexes, functioning as critical regulators of cell cycle at the G1/S-phase transition [40], [41]. Cell cycle checkpoint kinase 2 (CHK2) is known to inhibit CDC25C phosphatase and stabilize the p53 tumor suppressor protein, leading to cell cycle arrest in G1 [42]. Most interestingly, CENP-A silencing induced a G1 arrest of the cell cycle in HepG2 cells. These results suggest the antiproliferative effects of targeting CENP-A are linked to the upregulation of negative cell-cycle regulators including CHK2, P21waf1, and P27 Kip1. However, we found no significant relationship between CENP-A and P21waf1 in HCC samples by immunohistochemistry, reflecting a complex regulation of gene expression by CENP-A. Among the most downregulated genes by CENP-A silencing are SKP2, RAD51, and CCNG1. SKP2 is of particular interest, as it plays a key role in S-phase entry [43]. SKP2 has been shown to target P27 Kip1 and cyclin E for degradation and control the cell cycle at the G1-S transition [44]. Taken together, our data indicate that CENP-A affects HCC cell proliferation via the coordination of multiple positive and negative regulators of cell-cycle progression.

The P53 pathway has been shown to mediate cellular stress responses, inducing cell-cycle arrest, senescence, and apoptosis [34]. Our clinical data indicate that there is a significant correlation between CENP-A and P53 immunopositivity in HCC. The involvement of the P53 pathway in CENP-A action has been documented in a previous study [38], where P53 was found to be essential for the CENP-A-depletion-induced cellular senescence. To test whether the P53 pathway was involved in the effects of CENP-A on HCC cell apoptosis, we examined the expression of several key components of this pathway (i.e., P53, MDM2, Bcl-2, and Bax) in the HepG2 cells overexpressing or depleted CENP-A. The endogenous P53 inhibitor MDM2 and the anti-apoptotic protein Bcl-2 protein amounts were significantly elevated in CENP-A-overexpressing cells, while the expression of the pro-apoptotic protein Bax was compromised under the same condition. Conversely, silencing of CENP-A decreased the MDM2 and Bcl-2 protein levels but increased the Bax protein products. However, no significant alteration in the P53 expression was observed by manipulating CENP-A in HepG2 cells. These results collectively suggest that targeting CENP-A can induce HCC cell apoptosis, likely through a P53-independent pathway. A recent study has demonstrated that genetic SKP2 inactivation suppresses tumorigenesis by evoking a P53-independent cellular senescence [45], raising the possibility that in at least some HCC cells, CENP-A reduction promoted apoptosis through the inhibition of SKP2. Further investigations are needed to test this hypothesis.

In summary, CENP-A is frequently overexpressed in HCC and its overexpression correlates with tumor histological grade and P53 immunopositivity. RNAi-mediated CENP-A depletion suppresses HCC cell growth both in vitro and in vivo, blocks cell-cycle progression at the G1 phase, and promotes apoptosis. The anticancer effects of depleting CENP-A are likely mediated through the regulation of a large number of genes involved in cell-cycle control and apoptosis, including CHK2, P21waf1, P27 Kip1, SKP2, MDM2, Bcl-2, and Bax. These data suggest that CENP-A may be a potential therapeutic target for HCC.

## Materials and Methods

### Cell line and cell culture

HepG2 and SMMC-7721 cell lines (wild-type p53 and HBV negative) were purchased from Institute of Cellular Research, Chinese Academy of Science, Shanghai, China. Cells were routinely cultured as previously described [46].

### Human HCC samples

Tumor samples from resection specimens were collected from two consecutive cohorts of patients with HCC. Cohort A consisted of 20 patients, from whom fresh tumor samples coupled with adjacent nontumor liver tissues were obtained for analysis of CENP-A gene expression. After removal at surgery, all the fresh tissues were cut into small pieces, snap-frozen in liquid nitrogen immediately, and stored at −80°C until RNA or protein extraction. Cohort B comprised 80 HCC patients, whose paraffin-embedded tumor tissues represented on tissue microarrays were analyzed by immunohistochemistry. All tissues were obtained during surgeries in Changhai Hospital between August 2003 and October 2008 with prior patients' consent and approval from the Ethics Committee of Second Military Medical University. The excised samples were obtained within one hour after the operation from tumor tissues and the corresponding adjacent nontumor tissues 5 to 10 cm from the tumor. The patients of both cohorts were selected based on (a) distinctive pathologic diagnosis of HCC according to the World Health Organization histological classification of tumors of the liver and intrahepatic bile ducts (2000) [47], (b) receiving curative resection, defined as macroscopically complete removal of the neoplasm, and (c) availability of detailed clinicopathologic data. Patients with preoperative anticancer treatment or with evidence of other malignancies were excluded from the study. All patients were negative for serum HCV and HIV.

### RNA isolation, reverse transcription-polymerase chain reaction (RT-PCR), and qPCR

RT-PCR and qPCR analysis was performed as previously described [48], [49]. Briefly, total RNA was extracted from cells or tissues with an RNeasy mini kit (Qiagen, Germany) according to the manufacturer's protocol. Complementary DNA (cDNA) was synthesized from total RNA with the First-Strand cDNA Synthesis Kit for RT-PCR (Roche, Mannheim, Germany). RT-PCR amplification was achieved using Taq DNA Polymerase (AmpliTaq Gold; Roche Molecular Systems, Pleasanton, CA) at the following conditions: one cycle at 94°C for 5 min, 30 cycles at 95°C for 30 sec, 58°C for 45 sec, and 72°C for 1.5 min, and one cycle at 72°C for 10 min. CENP-A cDNA was amplified with primers: forward 5′-ACAAGGTTGGCTAAAGGA-3′ and reverse 5′-ATGCTTCTGCTGCCTCTT-3′ (178 bp). For controls, glyceraldehyde-3-phosphate dehydrogenase (GAPDH) was amplified in a parallel reaction, with the following primers: 5′-TCCACCACCCTGTTGCTGTA-3′ and 5′-ACCACAGTCCATGCCATCAC-3′ (452 bp). RT-PCR products were separated by electrophoresis on 1.5% agarose gels. The qPCR was performed using SYBR-green detection of PCR products in real time with the Light Cycler (Roche Diagnostics, Meylan, France). The cycling conditions were as follows: initial denaturation at 95°C for 10 min, and then 45 cycles of denaturation at 95°C for 5 s, annealing at 62°C for 20 s, and elongation at 72°C for 15 s. As an internal quantitative control, β-actin gene expression was determined with the primers: 5′-GAGCGGGAAATCGTGCGTGACATT-3′ and 5′-GATGGAGTTGAAGGTAGTTTCGTG-3′ (234 bp). Gene expression was analyzed using the Light Cycler software Version 3.5 (Roche Diagnostics), and the ratios of CENP-A to β-actin represented normalized relative levels of CENP-A expression [50]. A no-template negative control was also included in each experiment. Analyses of all samples were carried out in triplicate, and the mean values were calculated.

### Protein extraction and Western blotting analysis

Primary antibodies employed were: anti-CENP-A (ab13939, Abcam, Cambridge, UK; 1∶500), anti-GAPDH (Santa Cruz Biotech, Santa Cruz, CA; 1∶2000), anti-P53 (Do-1, sc-126, Santa Cruz Biotech; 1∶300), anti-P21waf1 (F-5, sc-6246, Santa Cruz Biotech; 1∶200), anti-SKP2 (F-5, sc-7163, Santa Cruz Biotech; 1∶200), anti-Bcl-2 (sc-7382, Santa Cruz Biotech; 1∶400), anti-Bax (sc-7480, Santa Cruz Biotech; 1∶300), anti-MDM2 (sc-965, Santa Cruz Biotech; 1∶400), anti-CHK2 (ab47433, Abcam, Cambridge, UK; 1∶500), anti-RAD51 (ab88572, Abcam, Cambridge, UK; 1∶500), and anti-cyclin G.1(sc-8016, Santa Cruz Biotech; 1∶300). Protein isolation of culture cells or tissues and Western blotting were carried out as previously described [46]. Briefly, culture cells or tissues were lysed in buffer containing 10 mmol/L Tris (pH 7.4), 1% sodium dodecyl sulfate (SDS), 1 mmol/L sodium orthovanadate, and complete protease inhibitors (Roche). The extraction was accomplished using the Klose method as described previously [51], [52]. Samples of the lysates (50 µg) were then separated using SDS-polyacrylamide gel electrophoresis (SDS-PAGE), transferred to polyvinylidene fluoride membranes (Millipore, Bedford, MA), and blotted with the primary antibodies. After the incubation with horseradish peroxidase-conjugated secondary antibody (Santa Cruz Biotech), the blots were visualized with enhanced chemiluminescence (sc-2048, Santa Cruz Biotech). The intensity of each band was measured using a Fluor-S MultiImager and Quantity-One software (Bio-Rad, Hercules, CA).

### Immunohistochemistry

Immunohistochemistry was performed by SP-9000 HistostainTM-plus kit (Zhongshan golden bridge Biotech, Beijing, China) as previously described [46]. The primary antibodies included anti-CENP-A (ab13939, Abcam; 1∶100), anti-Ki-67 (MIB-1, DAKO, Glostrup, Denmark; 1∶100), anti-P53 (Santa Cruz Biotech; 1∶50), and anti-P21waf1 (Santa Cruz Biotech; 1∶40). We defined that a less than 10% represented low, and more than 10% represented high levels of CENP-A and P21 waf1 expression [49]. P53 staining was considered positive when more than 5% nuclei of cells were immunopositive. The Ki-67 labeling index was calculated as the percentage of immunopositive of cells.

### Plasmid construction and transfection

Full-length human CENP-A (GenBank number: U14518) was amplified by PCR and cloned into the pcDNA3.0 vector (Invitrogen, Carlsbad, CA) for overexpression studies. The sequences of primers for CENP-A were as follows: forward 5′-GCTCTAGATCCCCAGAAGCCAGCCTTTC-3′ and reverse 5′-TAGAATTCCTCAGCCGAGTCCCTCCTCA-3′. For knockdown of CENP-A expression, three siRNAs were designed following the rules of Tuschl [53]. The siRNA sequences were as follows: CENP-A1, 5′-AAAGGAGATCCGAAAGCTTCA-3′ (corresponding to nucleotides 144 to 164), CENP-A2, 5′-AAATATGTGTTAAATTCACTC-3′ (corresponding to nucleotides 218 to 238), and CENP-A3, 5′-AAGCATTTCTAGTTCATCTCTTT-3′ (corresponding to nucleotides 296 to 318). The siRNA oligonucleotides were synthesized and inserted into the pSilencer 2.1-U6 vector (Ambion, Inc., Austin, TX). The integrity of all the constructs was confirmed by DNA sequencing. The plasmids were transfected into HepG2 cells using Lipofectamine 2000 (Invitrogen, Carlsbad, CA), according to the manufacture's protocol. The CENP-A1 and CENP-A2 siRNAs with high knockdown efficiency were used for the subsequent experiments. Stable transfectants were selected for 2 weeks in the presence of G418 (400 µg/mL). HepG2 cells stably transfected with CENP-A1 and CENP-A2 were named as pSil2.1-CENP-A1 and pSil2.1-CENP-A2, respectively.

### Proliferation assay

Transfected HepG2 cells were plated in 96-well microplates (1.5×10^3^ cells per well and six repeats per cell line). Cells were counted daily over a 7-day period by the CellTiter 96 Non-Radioactive Cell Proliferation Assay (Promega, Madison, WI) [46]. The dark-blue crystals of MTT-formazan were dissolved by shaking the plates at room temperature for 10 min and the absorbance was then measured on a Bio-Rad Microplate Reader at a test wavelength of 490 nm and a reference wavelength of 630 nm. Each growth curve showed the means and standard deviation (SD) of at least three independent experiments.

### Colony formation

Detailed experimental procedures were as described previously [46], [54]. Briefly, transfected HepG2 cells were plated in six-well plates at a density of 1,000 cells per well in routine culture medium. After 10 days, cells were washed with PBS, fixed in 10% methanol for 15 min, and stained in Giemsa for 20 min. Colonies consisting of >50 cells were scored. Each experiment was repeated three times.

### Cell cycle and apoptosis analysis

Cellular DNA content was analyzed by flow cytometry as described previously [46]. Briefly, HepG2 transfectants in logarithmic phase were harvested, fixed in ethanol, and incubated with 0.5 mg/ml of propidium iodide (PI) along with 0.1 mg/ml of RNase A (200 KU; Calbiochem, San Diego, CA), prior to cell-cycle analysis. Apoptosis was measured with an Annexin V/FITC kit (LHK601-100, Bender Medsystems, Austria) according to the manufacturer's protocol. Each assay was repeated three times.

### Tumorigenicity in nude mice

The tumorigenicity assay was performed as previously described [46]. Nude mice were purchased from the Experimental Animal Center of Shanghai, Shanghai, China. They were randomly divided into 4 groups (3 mice in each group), and were injected subcutaneously at a single site with 2×10^6^ cells stably transfected with pcDNA3.0-CENP-A, CENP-A1, or empty vectors or with untreated HepG2 cells. All experimental manipulations were undertaken in accordance with the National Institutes of Health Guide for the Care and Use of Laboratory Animals, with the approval of the Scientific Investigation Board of the Second Military Medical University, Shanghai, China. The mice were observed for 30 days for tumor formation and then sacrificed. The tumors were harvested and verified as being hepatocellular carcinoma by hematoxylin and eosin (HE) staining. The number and diameter of tumors were measured.

### RT^2^ Profiler™ PCR array

To profile the gene expression associated with cell-cycle regulation, we employed the Human Cell Cycle RT^2^ Profiler™ PCR Array (SuperArray, Frederick, MD). RNA isolation, DNase treatment, and RNA clean-up were performed according to the manufacturer's protocol (Qiagen, Hilden, Germany). The isolated RNA was reverse transcribed into cDNA using the RT^2^ First Strand Kit (Invitrogen). PCR was performed using the RT^2^ SYBR Green qPCR Master Mix (Invitrogen) on an ABI PRISM7900 instrument (Applied Biosystems, Foster City, CA). Data normalization was based on correcting all Ct values for the average Ct values of several constantly expressed housekeeping genes present on the array [55]. The analysis was completed by Shanghai KangChen Bio-tech Company, Shanghai, China. Each assay was conducted in triplicate.

### Statistical analysis

Data were presented as means±SD. All statistical calculations were carried out using SPSS.11 software (SPSS, Chicago, IL). The difference among the means of multiple groups was analyzed by one-way analysis of variance (ANOVA) followed by the Tukey test. A difference was defined as significant at *P*<0.05 in Student's t- test, one-way ANOVA, or Chi-square test. A correlation coefficient >0.5 or <−0.5 was considered significant for Spearman's correlation.

## Supporting Information

Table S1 **Positive and negative transcriptional regulation by CENP-A knock-down in hepatoma cells.**
(DOC)
