# Supplementary material for: ShRNA-Targeted Centromere Protein A Inhibits Hepatocellular Carcinoma Growth
Source: PLoS One. 2011 Mar 15;6(3):e17794. doi: 10.1371/journal.pone.0017794 (PMC3058037; doi:10.1371/journal.pone.0017794)
Supplement: Table S1 — Positive and negative transcriptional regulation by CENP-A knock-down in hepatoma cells. (DOC) [file pone.0017794.s001.doc]

**Table 2. Positive and negative transcriptional regulation by CENP-A knock-down in hepatoma cells**

| Position | RefSeq | Description | Symbol | Gene Name | Fold Difference Ratio |
| --- | --- | --- | --- | --- | --- |
| A01 | NM_005157 | C-abl oncogene 1, receptor tyrosine kinase | ABL1 | ABL/JTK7 | 0.53±0.11 |
| A02 | NM_013366 | Anaphase promoting complex subunit 2 | ANAPC2 | APC2 | 0.70±0.14 |
| A03 | NM_013367 | Anaphase promoting complex subunit 4 | ANAPC4 | APC4 | 0.98±0.09 |
| A04 | NM_004675 | DIRAS family, GTP-binding RAS-like 3 | DIRAS3 | ARHI/NOEY2 | **0.35±0.04** |
| A05 | NM_000051 | Ataxia telangiectasia mutated | ATM | AT1/ATA | 0.72±0.17 |
| A06 | NM_001184 | Ataxia telangiectasia and Rad3 related | ATR | FRP1/MEC1 | 0.63±0.13 |
| A07 | NM_004324 | BCL2-associated X protein | BAX | Bax zeta | 1.77±0.22 |
| A08 | NM_016567 | BRCA2 and CDKN1A interacting protein | BCCIP | TOK-1 | 1.04±0.19 |
| A09 | NM_000633 | B-cell CLL/lymphoma 2 | BCL2 | Bcl-2 | 0.68±0.24 |
| A10 | NM_001168 | Baculoviral IAP repeat-containing 5 (survivin) | BIRC5 | API4/EPR-1 | **0.39±0.05** |
| A11 | NM_007294 | Breast cancer 1, early onset | BRCA1 | BRCAI/BRCC1 | 0.74±0.18 |
| A12 | NM_000059 | Breast cancer 2, early onset | BRCA2 | BRCC2/FACD | 0.67±0.12 |
| B01 | NM_031966 | Cyclin B1 | CCNB1 | CCNB | **0.38±0.08** |
| B02 | NM_004701 | Cyclin B2 | CCNB2 | HsT17299 | 0.58±0.09 |
| B03 | NM_005190 | Cyclin C | CCNC | CycC | **0.45±0.07** |
| B04 | NM_053056 | Cyclin D1 | CCND1 | BCL1/D11S287E | 0.68±0.12 |
| B05 | NM_001759 | Cyclin D2 | CCND2 | KIAK0002 | 1.32±0.12 |
| B06 | NM_001238 | Cyclin E1 | CCNE1 | CCNE | 0.88±0.15 |
| B07 | NM_001761 | Cyclin F | CCNF | FBX1/FBXO1 | 0.68±0.17 |
| B08 | NM_004060 | Cyclin G1 | CCNG1 | CCNG | **0.27±0.06** |
| B09 | NM_004354 | Cyclin G2 | CCNG2 | Cyclin G2 | 0.73±0.14 |
| B10 | NM_001239 | Cyclin H | CCNH | CAK/p34 | 0.71±0.09 |
| B11 | NM_001240 | Cyclin T1 | CCNT1 | CCNT/CYCT1 | 0.76±0.12 |
| B12 | NM_001241 | Cyclin T2 | CCNT2 | FLJ90560 | **0.46±0.09** |
| C01 | NM_003903 | Cell division cycle 16 homolog (S. cerevisiae) | CDC16 | APC6 | 0.63±0.12 |
| C02 | NM_001786 | Cell division cycle 2, G1 to S and G2 to M | CDC2 | CDC28A/CDK1 | 0.59±0.19 |
| C03 | NM_001255 | Cell division cycle 20 homolog (S. cerevisiae) | CDC20 | CDC20A/p55CDC | 0.52±0.09 |
| C04 | NM_004359 | Cell division cycle 34 homolog (S. cerevisiae) | CDC34 | E2-CDC34/UBC3 | 1.07±0.18 |
| C05 | NM_001798 | Cyclin-dependent kinase 2 | CDK2 | p33(CDK2) | **0.47±0.04** |
| C06 | NM_000075 | Cyclin-dependent kinase 4 | CDK4 | CMM3/PSK-J3 | 0.56±0.04 |
| C07 | NM_003885 | Cyclin-dependent kinase 5, regulatory subunit 1 (p35) | CDK5R1 | CDK5P35/CDK5R | **0.49±0.07** |
| C08 | NM_016408 | CDK5 regulatory subunit associated protein 1 | CDK5RAP1 | C20orf34/C42 | 0.68±0.12 |
| C09 | NM_001259 | Cyclin-dependent kinase 6 | CDK6 | PLSTIRE | 1.42±0.09 |
| C10 | NM_001799 | Cyclin-dependent kinase 7 | CDK7 | CAK1/CDKN7 | 0.78±0.14 |
| C11 | NM_001260 | Cyclin-dependent kinase 8 | CDK8 | K35 | 0.60±0.12 |
| C12 | NM_000389 | Cyclin-dependent kinase inhibitor 1A (p21, Cip1) | CDKN1A | CAP20/CDKN1 | **8.33±0.13** |
| D01 | NM_004064 | Cyclin-dependent kinase inhibitor 1B (p27, Kip1) | CDKN1B | CDKN4/KIP1 | **2.08±0.05** |
| D02 | NM_000077 | Cyclin-dependent kinase inhibitor 2A (melanoma, p16, inhibits CDK4) | CDKN2A | ARF/CDK4I | 0.77±0.12 |
| D03 | NM_004936 | Cyclin-dependent kinase inhibitor 2B (p15, inhibits CDK4) | CDKN2B | CDK4I/INK4B | 0.51±0.07 |
| D04 | NM_005192 | Cyclin-dependent kinase inhibitor 3 (CDK2-associated dual specificity phosphatase) | CDKN3 | CDI1/CIP2 | 1.22±0.11 |
| D05 | NM_001274 | CHK1 checkpoint homolog (S. pombe) | CHEK1 | CHK1 | 0.54±0.15 |
| D06 | NM_007194 | CHK2 checkpoint homolog (S. pombe) | CHEK2 | CDS1/CHK2 | **4.55±0.08** |
| D07 | NM_001826 | CDC28 protein kinase regulatory subunit 1B | CKS1B | CKS1/PNAS-16 | 0.78±0..28 |
| D08 | NM_001827 | CDC28 protein kinase regulatory subunit 2 | CKS2 | CKSHS2 | 0.55±0.08 |
| D09 | NM_003592 | Cullin 1 | CUL1 | MGC149834 | 1.21±0.14 |
| D10 | NM_003591 | Cullin 2 | CUL2 | MGC131970 | 1.01±0.19 |
| D11 | NM_003590 | Cullin 3 | CUL3 | Cullin-Cul3 | 1.61±0.24 |
| D12 | NM_004399 | DEAD/H (Asp-Glu-Ala-Asp/His) box polypeptide 11 (CHL1-like helicase homolog, S. cerevisiae) | DDX11 | CHL1/CHLR1 | **0.36±0.06** |
| E01 | NM_004945 | Dynamin 2 | DNM2 | CMTDI1/CMTDIB | 1.11±0.28 |
| E02 | NM_001950 | E2F transcription factor 4, p107/p130-binding | E2F4 | E2F-4 | 0.54±0.12 |
| E03 | NM_001924 | Growth arrest and DNA-damage-inducible, alpha | GADD45A | DDIT1/GADD45 | 0.54±0.08 |
| E04 | NM_005316 | General transcription factor IIH, polypeptide 1, 62kDa | GTF2H1 | BTF2/TFIIH | 0.52±0.11 |
| E05 | NM_016426 | G-2 and S-phase expressed 1 | GTSE1 | B99 | 0.72±0.12 |
| E06 | NM_016323 | Hect domain and RLD 5 | HERC5 | CEB1/CEBP1 | 0.85±0.23 |
| E07 | NM_004507 | HUS1 checkpoint homolog (S. pombe) | HUS1 | Hus1 | 0.59±0.26 |
| E08 | NM_014708 | Kinetochore associated 1 | KNTC1 | ROD | 0.92±0.25 |
| E09 | NM_002266 | Karyopherin alpha 2 (RAG cohort 1, importin alpha 1) | KPNA2 | IPOA1/QIP2 | 0.97±0.14 |
| E10 | NM_002358 | MAD2 mitotic arrest deficient-like 1 (yeast) | MAD2L1 | HSMAD2/MAD2 | 0.63±0.19 |
| E11 | NM_006341 | MAD2 mitotic arrest deficient-like 2 (yeast) | MAD2L2 | MAD2B/REV7 | 0.96±0.17 |
| E12 | NM_004526 | Minichromosome maintenance complex component 2 | MCM2 | BM28/CCNL1 | 1.57±0.11 |
| F01 | NM_002388 | Minichromosome maintenance complex component 3 | MCM3 | HCC5/P1-MCM3 | **0.37±0.05** |
| F02 | NM_005914 | Minichromosome maintenance complex component 4 | MCM4 | CDC21/CDC54 | **0.42±0.09** |
| F03 | NM_006739 | Minichromosome maintenance complex component 5 | MCM5 | CDC46/P1-CDC46 | **0.35±0.07** |
| F04 | NM_002417 | Antigen identified by monoclonal antibody Ki-67 | MKI67 | KIA/Ki-67 | 0.82±0.11 |
| F05 | NM_002431 | Menage a trois homolog 1, cyclin H assembly factor (Xenopus laevis) | MNAT1 | MAT1/RNF66 | 0.95±0.24 |
| F06 | NM_005590 | MRE11 meiotic recombination 11 homolog A (S. cerevisiae) | MRE11A | ATLD/HNGS1 | 0.58±0.14 |
| F07 | NM_002485 | Nibrin | NBN | AT-V1/AT-V2 | 0.78±0.20 |
| F08 | NM_182649 | Proliferating cell nuclear antigen | PCNA | MGC8367 | 0.66±0.14 |
| F09 | NM_002853 | RAD1 homolog (S. pombe) | RAD1 | HRAD1/REC1 | **0.43±0.07** |
| F10 | NM_002873 | RAD17 homolog (S. pombe) | RAD17 | CCYC/HRAD17 | 0.57±0.15 |
| F11 | NM_002875 | RAD51 homolog (RecA homolog, E. coli) (S. cerevisiae) | RAD51 | BRCC5/HRAD51 | **0.21±0.09** |
| F12 | NM_004584 | RAD9 homolog A (S. pombe) | RAD9A | RAD9 | 1.32±0.19 |
| G01 | NM_000321 | Retinoblastoma 1 (including osteosarcoma) | RB1 | OSRC/RB | 1.14±0.09 |
| G02 | NM_002894 | Retinoblastoma binding protein 8 | RBBP8 | CTIP/RIM | 1.13±0.12 |
| G03 | NM_002895 | Retinoblastoma-like 1 (p107) | RBL1 | CP107/PRB1 | **0.46±0.08** |
| G04 | NM_005611 | Retinoblastoma-like 2 (p130) | RBL2 | P130/Rb2 | 0.57±0.08 |
| G05 | NM_002947 | Replication protein A3, 14kDa | RPA3 | REPA3 | 0.56±0.09 |
| G06 | NM_013376 | SERTA domain containing 1 | SERTAD1 | SEI1/TRIP-Br1 | 1±0.15 |
| G07 | NM_005983 | S-phase kinase-associated protein 2 (p45) | SKP2 | FBL1/FBXL1 | **0.22±0.05** |
| G08 | NM_003352 | SMT3 suppressor of mif two 3 homolog 1 (S. cerevisiae) | SUMO1 | DAP-1/GMP1 | 0.80±0.15 |
| G09 | NM_007111 | Transcription factor Dp-1 | TFDP1 | DP1/DRTF1 | **0.31±0.04** |
| G10 | NM_006286 | Transcription factor Dp-2 (E2F dimerization partner 2) | TFDP2 | DP2/Dp-2 | 0.65±0.09 |
| G11 | NM_000546 | Tumor protein p53 | TP53 | LFS1/TRP53 | 1.55±0.14 |
| G12 | NM_003334 | Ubiquitin-like modifier activating enzyme 1 | UBA1 | A1S9/A1S9T | 0.61±0.15 |
| H01 | NM_004048 | Beta-2-microglobulin | B2M | B2M | 0.89±0.21 |
| H02 | NM_000194 | Hypoxanthine phosphoribosyltransferase 1 (Lesch-Nyhan syndrome) | HPRT1 | HGPRT/HPRT | 1.05±0.24 |
| H03 | NM_012423 | Ribosomal protein L13a | RPL13A | RPL13A | 0.51±0.15 |
| H04 | NM_002046 | Glyceraldehyde-3-phosphate dehydrogenase | GAPDH | G3PD/GAPD | 1.07±0.11 |
| H05 | NM_001101 | Actin, beta | ACTB | PS1TP5BP1 | 0.41±0.14 |
